# De Novo Genome Assembly and Phylogenetic Analysis of *Cirsium nipponicum*

**DOI:** 10.3390/genes15101269

**Published:** 2024-09-27

**Authors:** Bae Young Choi, Jaewook Kim, Hyeonseon Park, Jincheol Kim, Seahee Han, Ick-Hyun Jo, Donghwan Shim

**Affiliations:** 1 School of Liberal Arts and Sciences, Korea National University of Transportation, Chungju 27469, Republic of Korea; baeyoung@ut.ac.kr; 2 Department of Biology Education, Korea National University of Education, Cheongju 28173, Republic of Korea; jwkim@knue.ac.kr; 3 Department of Biological Sciences, Chungnam National University, Daejeon 34134, Republic of Korea; bhs2880@gmail.com; 4 Department of Crop Science and Biotechnology, Dankook University, Cheonan 31116, Republic of Korea; papirus1991@naver.com; 5 Division of Botany, Honam National Institute of Biological Resources, Mokpo 58762, Republic of Korea; seahee113@hnibr.re.kr; 6 Center for Genome Engineering, Institute for Basic Science, Daejeon 34126, Republic of Korea

**Keywords:** genome assembly, comparative genomics, thistle, *Cirsium nipponicum*

## Abstract

**Background: ***Cirsium nipponicum*, a pharmaceutically valuable plant from the Asteraceae family, has been utilized for over 2000 years. Unlike other thistles, it is native to East Asia and found exclusively on Ulleung Island on the Korea Peninsula. Despite its significance, the genome information of *C. nipponicum* has remained unclear. **Methods:** In this study, we assembled the genome of *C. nipponicum* using both short reads from Illumina sequencing and long reads from Nanopore sequencing. **Results:** The assembled genome is 929.4 Mb in size with an N50 length of 0.7 Mb, covering 95.1% of BUSCO core groups listed in edicots_odb10. Repeat sequences accounted for 70.94% of the assembled genome. We curated 31,263 protein-coding genes, of which 28,752 were functionally annotated using public databases. Phylogenetic analysis of 11 plant species using single-copy orthologs revealed that *C. nipponicum* diverged from *Cynara cardunculus* approximately 15.9 million years ago. Gene family evolutionary analysis revealed significant expansion and contraction in genes involved in abscisic acid biosynthesis, late endosome to vacuole transport, response to nitrate, and abaxial cell fate specification. **Conclusions:** This study provides a reference genome of *C. nipponicum*, enhancing our understanding of its genetic background and facilitating an exploration of genetic resources for beneficial phytochemicals.

## 1. Introduction

*Cirsium nipponicum* (*C. nipponicum*) is a perennial flowering plant in the Asteraceae family, renowned for its diverse species with medicinal properties and distributed in East Asia. Unlike other thistle species in Korea, *C. nipponicum* has minimal thorns on its leaves and is found exclusively on Ulleung Island, an oceanic volcanic island in the Korean Peninsula [1]. Phytochemicals in *C. nipponicum*, such as polyphenols and flavonoids, exhibit antioxidant and anti-inflammatory activities, making them valuable for therapeutic purposes [2,3]. Of particular interest is the high accumulation of silymarin in the fruits of thistle plants [4]. Silymarin, a complex mixture of flavonoids and flavonolignans, has been extensively studied for its medicinal effects, particularly in the treatment of liver disorders [5,6]. Despite the pharmaceutical importance of thistle species, to date only one genomic sequence of a thistle, *Silybum marianum* (L.) Gaertn., has been studied, to the best of our knowledge [7].

Oceanic islands are important ecosystems for studying the evolutionary history of organisms, due to their spatial and temporal confinement [8]. Ulleung Island, a biodiversity hotspot in Korea, is home to many species [9]. Anagenetic speciation, the evolutionary change within a lineage, has been extensively studied on Ulleung Island [10], highlighting its significance in evolutionary research for diverse species. In Korea, there are eight thistle species of the genus *Cirsium*, namely *C. lineare*, *C. vlassovianum*, *C. setidens*, *C. pendulum*, *C. japonicum*, *C. schantarense*, *C. rhinoceros*, and *C. nipponicum* [11]. However, the genetic resources of these thistle species remain largely unexplored. Recent sequencing of six *Cirsium* plastomes suggested that *C. nipponicum* was introduced from the north Eurasian region and evolved independently on Ulleung Island, distinct from other mainland Korean thistle species [1], suggesting that Ulleung Island *C. nipponicum* might possess unique genetic characteristics that could enhance our understanding of thistle species evolution.

In this study, we assembled the genome of *C. nipponicum* using a combination of Illumina short reads and Nanopore long reads. We annotated 31,263 protein-coding genes using RNA-seq data and protein homology information. Orthologous gene identification and phylogenetic analysis of the *C. nipponicum* genome, along with the genomes of 10 other representative plants, revealed that *C. nipponicum* formed a monophyletic clade with *C. cardunculus* and diverged approximately 15.9 million years ago (Mya). Furthermore, we found that genes related to abscisic acid biosynthesis, late endosome to vacuole transport, response to nitrate, and abaxial cell fate specification in *C. nipponicum* exhibited both significant expansion and contraction compared to the other species. The genomic resources, gene structure, and comparative genomic analyses of *C. nipponicum* will improve our understanding of thistle species.

## 2. Material and Methods

### 2.1. Plant Materials, DNA, and RNA Extraction

**Figure 1:**
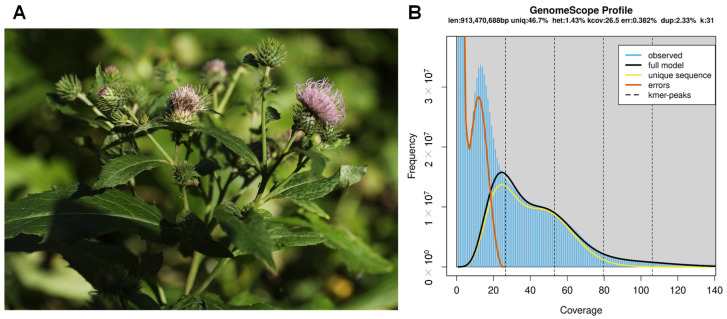
Morphology and K-mer analysis of *C. nipponicum*. (**A**) *C. nipponicum* plant in the reproductive stage on Ulleung Island, displaying flowers with emerging petals. (**B**) The genome size was estimated as 913 Mb with 1.43% heterozygosity using 31-mer.

Whole plants of *C. nipponicum* were collected from Ulleung Island (Figure 1A). Healthy leaves were used for whole genome sequencing (WGS). Total genomic DNA was extracted using a Wizard^®^ Genomic DNA Purification kit (Promega, Madison, WI, USA). Total RNA was extracted from leaves, roots, stems, and flowers using a SmartGene Plant RNA Extraction kit (SmartGene, Daejeon, Republic of Korea). The quality and quantity of the DNA and RNA samples were evaluated using a 4150 TapeStation (Agilent, Santa Clara, CA, USA) and a Qubit 4.0 Fluorometer (Invitrogen Ltd., Paisley, UK), respectively.

### 2.2. Short-Read Illumina Sequencing

The genomic DNA library was prepared using an xGen™ DNA Lib Prep EZ kit (Integrated DNA Technologies, Coralville, LA, USA). All RNA samples with RIN values > 7 were pulled, and mRNA was enriched using a Poly(A) RNA Selection Kit (Lexogen, Vienna, Austria). The enriched mRNA was subjected to 150 bp paired-end library preparation using an xGen™ RNA Lib Prep kit (Integrated DNA Technologies, Coralville, LA, USA). The generated Illumina libraries were sequenced on an Illumina NovaSeq 6000 platform (Illumina, San Diego, CA, USA).

### 2.3. Long-Read Nanopore Sequencing

Genomic DNA samples with a DNA integrity number > 8.0 were subjected to long read Oxford Nanopore Technologies (ONT) library preparation using a SQK-LSK110 kit (ONT, Oxford, UK). The pulled RNA was subjected to long-read cDNA library preparation using a PCS-109 kit (ONT, Oxford, UK). The long-read libraries were sequenced on the ONT MinION platform (ONT, Oxford, UK) according to the manufacturer’s instructions.

### 2.4. Genome Assembly

Paired-end short WGS reads were trimmed using Trimmomatic v. 0.39 with leading 15; trailing 15; and min. length 150 options [12]. To estimate genome size and heterozygosity of *C. nipponicum*, the *k*-mer frequency of cleaned reads was analyzed using the JELLYFISH v. 2.3.0 [13] and GenomeScope v. 2.0 [14].

Error correction for long WGS reads were conducted using Ratatosk v. 0.7.6, utilizing paired-end short WGS reads [15]. The error-corrected long reads were then subject to de novo genome assembly using NextDenovo v. 2.5.0 [16]. Both long and short reads were used to polish the genome using NextPolish v. 1.4.0 [17]. Haplotypic duplicated genome sequences were removed using Purge-dups software v. 1.2.5 [18]. The statistics of the assembled genome were analyzed using QUAST, and the completeness of the genome assembly was evaluated using BUSCO v 5.2.2 with 2326 single-copy orthologs from the Eudicots odb10 database [19,20].

### 2.5. Genome Annotation

Transposable elements (TEs) and repeat sequences were annotated in two steps. First, a de novo repeat library was built using RepeatModeler v. 2.0.4 [21], and Repbase database was downloaded [22]. Second, the TEs and repeat sequences in the library and database were analyzed and annotated using RepeatMasker v. 4.1.5 (https://www.repeatmasker.org/RepeatMasker/, accessed on 16 June 2022).

The BRAKER3 pipeline [23] was used to annotate the protein-coding genes of *C. nipponicum* with three different hints: short-read RNA-seq data, protein homology information, and long-read isoform sequencing (IsoSeq) data. PRINSEQ-lite v. 0.20.4 was used to trim and filter short RNA-seq reads with the following parameters: min len 50; min qual score 10; min qual mean 20; derep 14; trim qual left 20; trim qual right 20 [24]. Cleaned reads were aligned to the genome using HISAT2 v. 2.2.1 [25], and the resulting mapping files were supplied to the BRAKER3 pipeline along with protein sequences from four plant species (*Arabidopsis thaliana*, *Carthamus tinctorius*, *Centaurea solstitialis*, *Cynara cardunculus*). In this pipeline, protein sequence data and RNA-seq data were processed by DIAMOND and Stringtie2, respectively, to serve as hints for gene prediction [26,27]. The resulting hints were used for training GeneMark-ETP and AUGUSTUS to predict genes [28,29]. Errors in long-read IsoSeq data were corrected by Ratatosk v. 0.7.6 using paired-end short RNA-seq data [15]. Error-corrected Isoseq data were aligned to the genome using Minimap2 v. 2.25 [30] and redundant isoforms were collapsed using Cupcake (https://github.com/Magdoll/cDNA_Cupcake, accessed on 23 March 2016). GeneMarkS-T was employed to predict genes in the unique isoforms [31]. Finally, all predicted gene models were combined to produce non-redundant and consensus gene sets using TSEBRA [23].

Functional annotation of protein-coding genes were conducted using EnTAP v. 1.1.1 [32] with two methods. First, protein sequences of protein-coding genes were subjected to a BLASTp analysis against the NCBI RefSeq database [33] and Uniprot database using DIAMOND [26,34]. Second, genes were annotated with KEGG terms and GO terms using eggNOG-mapper [35].

Transfer RNA (tRNA) genes were annotated using tRNAscan-SE v. 2.0.12 with eukaryote parameters [36]. Ribosomal RNA (rRNA) and its subunits were identified using Barrnap v. 0.9 in Eukaryotic mode [37]. To detect microRNA (miRNA) and small nuclear RNA (snRNA), sequence analysis was conducted by comparing against the Rfam database using Infernal’s cmscan v. 1.1.5 [38,39].

### 2.6. Phylogenetic Analyses

Gene families for the eleven plant species listed in Table S3 were clustered using OrthoFinder v. 2.5.5 with the -M msa option [40]. The resulting rooted species tree from a concatenated multiple sequence alignment of single-copy orthologs was used to infer the divergence times of *C. nipponicum* using MCMCtree implemented in PAML v. 4.10.7 with the following parameters: JC69 model, burnin 5,000,000; sampfreq 30; and nsample 10,000,000 [41]. The calibration time points were obtained from the TimeTree database (https://www.timetree.org, accessed on 23 March 2023) using nwkit [42]. We repeated the divergence time analysis, and the convergence of the two independent analyses was evaluated by calculating Pearson’s correlation coefficient (Figure S1). The phylogenetic tree with divergence times was visualized using MCMCtreeR v. 1.1 [43].

### 2.7. Gene Family Expansion and Contraction Analyses

To predict the contraction and expansion of gene families in *C. nipponicum* relative to their ancestors, the birth and death models were used to estimate the numbers of ancestral gene families based on orthogroup clustering and the phylogenetic tree with divergence times using CAFE5 v. 1.1 [44]. Gene families with a *p* value < 0.001 were defined as significantly expanded or contracted gene families. Gene Ontology (GO) enrichment analyses for significantly expanded and contracted gene families were conducted using topGO v. 2.52.0 [45]. GO terms with a *p*-value < 0.05 were summarized and visualized using REVIGO with default options [46].

### 2.8. Identification of Gene Duplications

To identify homologous gene pairs between four plants (*C. nipponicum*, *C. cardunculus*, *C. solstitialis*, *C. tinctorius*), protein sequences were subjected to BLASTp analysis using DIAMOND v. 2.1.9 with an evalue < 1 × 10^−5^ [26]. The homologous gene pairs were used to identify collinear blocks using MCScanX. The synonymous substitution rates (Ks) were calculated for each gene pairs using the add_ka_and_ks_to_collinearity.pl script implemented in MCScanX v. 1.1.11 [47].

## 3. Results and Discussion

### 3.1. Genome Sequencing and Assembly

We assembled the *C. nipponicum* genome using both the Illumina and ONT sequencing platforms. This approach generated approximately 128 Gb of short WGS reads, 89 Gb of long WGS reads with an N50 of 16 Kb, 21 Gb of short RNA-seq reads, and 6.3 Gb of long IsoSeq reads. We performed the K-mer analysis on the short WGS reads to estimate the genome size of *C. nipponicum*. Using a k-mer size of 31, the genome was estimated to be 913 Mb in size with a heterozygosity of 1.43% (Figure 1B).

The initial genome assembly using long WGS reads resulted in a genome size of 1487.8 Mb, comprising 5675 contigs with an N50 of 0.4 Mb (Table 1). This assembly size was larger than the genome size estimated from the K-mer analysis. To refine the assembly, we removed haplotypic duplicated genome sequences in the draft assembly, resulting in a purged genome of 929.4 Mb in length, consisting of 2199 contigs with an N50 of 0.7 Mb (Table 1). Notably, this genome size is the smallest reported within the *Cirsium* genus, as a comprehensive genome size estimate for 19 *Cirsium* species, excluding *C. nipponicum*, using flow cytometry revealed genome sizes in this genus ranging approximately from 1046 Mb to 5245 Mb [48,49,50].

To assess the completeness of the assembled genome, we searched for 2326 single-copy orthologs from the Eudicots odb10 database. BUSCO analysis indicated that 95.1% of the core genes were completely captured in the assembled genome, with 85.8% being single-copy genes and 9.3% duplicated genes. The percentages of fragmented and missing core genes were 0.8% and 4.1%, respectively (Table 2). The high BUSCO completeness score, along with the consistency between the genome size obtained from the assembly and the estimated size from K-mer analysis, implies a high-quality and complete *C. nipponicum* genome assembly.

### 3.2. Repeat Sequence and Gene Prediction

In the assembled genome of *C. nipponicum*, a total of 659.3 Mb sequences were identified as repetitive elements, accounting for 70.94% of the genome (Table S1). The high portion of repetitive elements in the *C. nipponicum* genome is consistent with findings in most plant genomes, where repetitive DNA constitutes a significant portion of the total genomic content [51]. The most abundant class of repetitive elements was long terminal repeat (LTR) elements, comprising 344.9 Mb (37.11%) of the genome. The substantial presence of LTR elements suggests that the large genome size of *C. nipponicum* might be due to the accumulation of these elements, since their accumulation plays a key role in genome size expansion in some plants [52,53]. Unclassified repeats were the next most abundant, accounting for 263.8 Mb (28.39%). Interspersed repeats comprised 637.6 Mb (68.61%), while non-interspersed repeats comprised 21.6 Mb (2%).

We identified and curated a total of 31,263 protein-coding genes with 5596 isoforms in the assembled genome. The average length of the primary transcripts was 1203 bp. Functional annotation of these protein-coding genes was conducted using multiple databases, including Refseq, Uniprot, and eggNOG database. Overall, 28,752 genes (91.97%) were functionally annotated in at least one of these databases (Table S2). We further identified various non-coding RNA structures in the *C. nipponicum* genome, which included 771 rRNA, 1137 tRNA, 159 miRNA, and 1907 snRNA genes (Table 3).

### 3.3. Comparative Genomic and Phylogenetic Analyses

**Figure 2:**
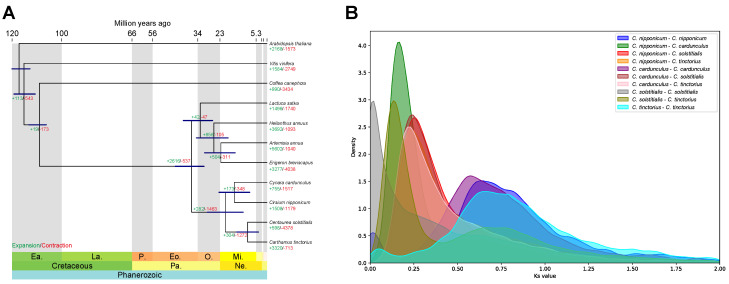
Comparative genomics analysis of *C. nipponicum* and other plant species. (**A**) Phylogenetic relationships of 11 plant species with single-copy orthologs identified using OrthoFinder. The divergence times (in millions of years ago) were estimated using MCMCTree, and the blue bars indicate highest posterior density intervals of at least 95%. The numbers in green and red indicate the expanded and contracted gene families relative to the most recent common ancestor. Ea, early; La, late; Pa, Paleogene; Ne, Neogene; P, Paleocene; Eo, Eocene; O, Oligocene; Mi, Miocene. (**B**) Distribution of the synonymous substitution rates (Ks) for pairs of paralogs and orthologs in the four plants (*C. nipponicum*, *C. cardunculus*, *C. solstitialis*, and *C. tinctorius*).

The evolutionary relationships within the green plant lineage and the phylogenetic position of *C. nipponicum* within the Asteraceae family were investigated through the analysis of primary protein-coding genes from 11 plant species using OrthoFinder [40]. We obtained 32,950 orthologous gene groups among these species. A phylogenetic tree was constructed using single-copy orthologous genes from these 11 plant species, and the divergence times were estimated (Figure 2A). Our phylogenetic analysis revealed that *C. nipponicum*, *C. cardunculus*, *C. solstitialis*, and *C. tinctorius* were grouped together, indicating that these species are closely related. These four species diverged from a common ancestor approximately 36.86 Mya. Specifically, *C. nipponicum* formed a monophyletic clade with *C. cardunculus*, which diverged from their common ancestor approximately 15.9 Mya.

To investigate species diversification and gene duplication events in *C. nipponicum*, we conducted Ks distribution analysis using paralogs and orthologs of *C. nipponicum* and its three closest relatives. The comparison of Ks distributions of the orthologs between *C. nipponicum* and *C. cardunculus* with those between *C. nipponicum* and *C. solstitialis* suggested that *C. nipponicum* diverged from *C. cardunculus* earlier than from *C. solstitialis*. This finding is consistent with the phylogenetic analysis and estimated divergence times (Figure 2A). The Ks distribution for paralogous genes in *C. nipponicum* showed a major peak at a Ks value of ~0.65, suggesting that gene duplication events occurred after the divergence of *C. nipponicum*.

**Figure 3:**
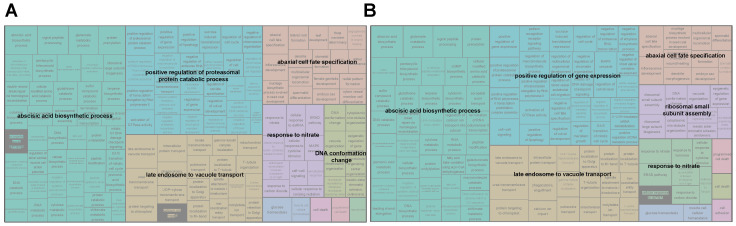
GO enrichment analysis of significantly expanded and contracted gene families in *C. nipponicum.* The GO enrichment analysis was performed on the 1760 expanded (**A**) and 365 contracted (**B**) gene families using topGO. The GO terms with a *p*-value < 0.05 listed in Tables S4 and S5 were further analyzed using REVIGO to identify representative enriched GO terms. The size of each rectangle in the treemap corresponds to the *p*-value of each GO term.

Using the birth and death model, we identified 1508 gene families in *C. nipponicum* that underwent expansion and 1179 gene families that underwent contraction relative to the most recent common ancestor of *C. nipponicum* and *C. cardunculus* (Figure 2A). GO enrichment analysis revealed that the significantly expanded gene families were mainly enriched in the positive regulation of the proteasomal protein catabolic process and DNA conformation change, while the significantly contracted gene families were primarily involved in the positive regulation of gene expression and ribosomal small subunit assembly (Figure 3). Notably, gene families involved in abscisic acid biosynthesis, late endosome to vacuole transport, response to nitrate, and abaxial cell fate specification exhibited both significant expansion and contraction.

In conclusion, the combination of the Illumina and ONT sequencing technologies, followed by rigorous assembly and polishing steps, has enabled the production of a high-quality genome assembly for *C. nipponicum*. The characterization of repeat sequences and the detailed annotation of protein-coding genes in the *C. nipponicum* genome provide a rich resource for future genetic and functional studies. Notably, the identification of genes involved in biosynthesis of silymarin, as well as potential biomarkers for silymarin production, could contribute to enhancing the yield of thistle-derived pharmaceutical products, which are promising phytochemicals for the treatment of liver-related diseases. Furthermore, our comparative genomic and phylogenetic analyses provide valuable insights into the evolutionary history of *C. nipponicum*. Its relationship with other species, coupled with significant gene family expansions and contractions, highlights the dynamic evolutionary processes shaping the genome of *C. nipponicum*. This genomic information enhances our understanding for comparative genomics and biotechnological applications using thistle species.

## Figures and Tables

**Table 1:** Statistics of the genome assembly and annotations.

Genome Assembly	Draft	Purge Haplotigs
Genome size (Mb)	1487.8	929.4
Number of contigs	5675	2199
N50 (bp)	421,852	700,963
GC contents (%)	36.01	35.76

**Table 2:** Statistics for genome assessment using BUSCO (edicots).

		Number of BUSCOs (%)
Complete		2212 (95.1)
	Complete and single-copy	1995 (85.8)
	Complete and duplicated	217 (9.3)
Fragmented		19 (0.8)
Missing		95 (4.1)

**Table 3:** Statistics of non-coding RNA in *C. nipponicum* genome.

Type	Copy	Average Length (bp)	Total Length (bp)
rRNA	771	139.66	107,681
tRNA	1137	76.00	86,409
miRNA	159	120.81	19,208
snRNA	1907	113.67	216,769

## Data Availability

All sequencing data generated in this study were deposited to the SRA database at the National Center for Biotechnology Information under the accession number PRJNA1127082. The assembled genome sequences and annotations are available at Figshare [10.6084/m9.figshare.26927092].

## Supplementary Materials

The following supporting information can be downloaded at https://www.mdpi.com/article/10.3390/genes15101269/s1: Figure S1: Convergence plot of two independent divergence time analyses using MCMCtree; Table S1: The statistics of repeat sequences; Table S2: Functional annotation of protein-coding genes using EnTAP; Table S3: The species used for phylogenetic analysis; Table S4: GO enrichment analysis of expanded gene families; Table S5: GO enrichment analysis of contracted gene families.

## Abbreviations

BUSCO	Benchmarking universal single-copy orthologs
BLAST	Basic local alignment search tool
Mb	Megabase pairs
IsoSeq	Isoform sequencing
ONT	Oxford Nanopore Technologies
TEs	Transposable elements
GO	Gene Ontology
Ks	Synonymous substitution rates
WGS	Whole genome sequencing
LTR	Long terminal repeat
Mya	Million years ago
