# Supplementary material for: De Novo Genome Assembly and Phylogenetic Analysis of Cirsium nipponicum
Source: Genes (Basel). 2024 Sep 27;15(10):1269. doi: 10.3390/genes15101269 (PMC11507141; doi:10.3390/genes15101269)
Supplement: Supplementary file 1 [file genes-15-01269-s001.zip › Supplementary figure S1.pdf]

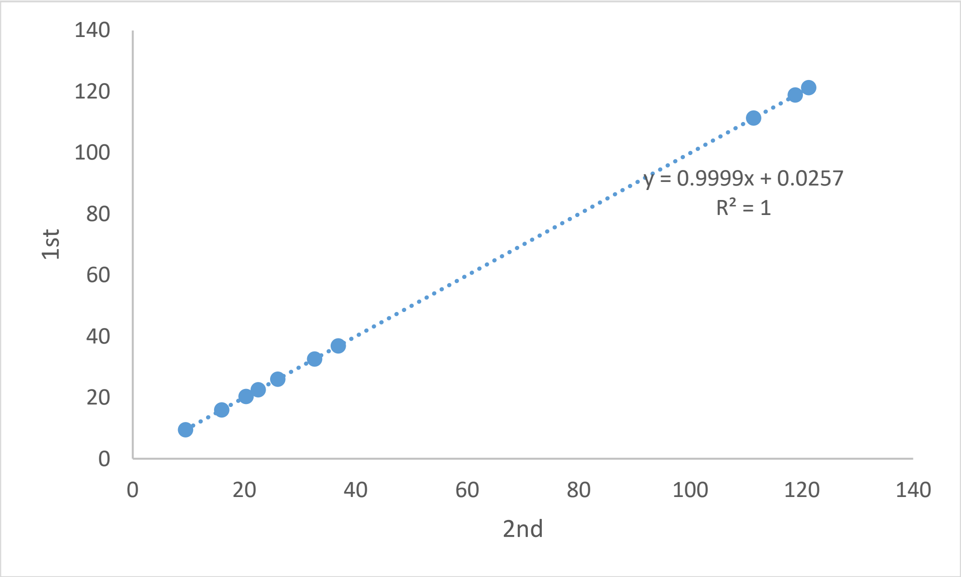

Supplementary Figure S1. Convergence plot of two independent divergence time analyses using MCMCtree.
